# Supplementary material for: A Touchscreen-Based, Multiple-Choice Approach to Cognitive Enrichment of Captive Rhesus Macaques (Macaca mulatta)
Source: Animals (Basel). 2023 Aug 24;13(17):2702. doi: 10.3390/ani13172702 (PMC10486349; doi:10.3390/ani13172702)
Supplement: Supplementary file 1 [file animals-13-02702-s001.zip › animals-2557155-supplementary.pdf]

Supplementary material for:

A Touchscreen-Based, Multiple-Choice Approach to Cognitive Enrichment of Captive Rhesus Macaques (*Macaca mulatta*)

Antonino Calapai [1,2,*], Dana Pfefferle [1,2], Lauren C. Cassidy [1,2,3], Anahita Nazari [1], Pinar Yurt [1,2], Ralf R. Brockhausen [1] and Stefan Treue [1,2,4]

[1] Cognitive Neuroscience Laboratory, German Primate Center, 37077 Goettingen, Germany
[2] Leibniz-Science Campus Primate Cognition, 37077 Goettingen, Germany
[3] Population and Behavioral Health Services, California National Primate Research Center, University of California, Davis, CA 95817, USA
[4] Faculty for Biology and Psychology, Goettingen University, 37073 Goettingen, Germany
* Correspondence: acalapai@dpz.eu

Further task preference analysis results

We determined if adult male rhesus macaques exhibited task preferences in a Multiple Choice Interface (MCI) experiment. Here, group- or pair-housed monkeys were presented with a choice between three screen buttons associated with a different task: viewing a random picture from a predetermined set (i.e., "picture task"), touch a stationary stimulus (i.e., "static task"), touching a moving stimulus (i.e., "dynamic task"). The static and dynamic tasks were rewarded with a small juice reward for touching the stimulus correctly. Overall, we conducted five to six sessions per social group (two to four adult male rhesus macaques), resulting in 68118 trials over 810 hours (median per session: 8.35 h; IQR: 0.36 h) of exposure.

Task selection behavior was examined using a categorical Bayesian Generalized Linear Mixed Model (described in the main text). To aid interpretation of the overall findings, we calculated the proportion of posterior samples that fell on the same side of 0 as the mean (Pr) to aid in the interpretation of whether the predictor variables substantially affected choice or trial outcome behavior. As the Pr ranges from 0.5 to 1.0, a Pr of 1.0 indicates the direction (negative or positive) of a predictor's effect, whereas a Pr of 0.5 indicates an effect centered around 0 (i.e., no effect on response variable).

Overall, we found evidence that the monkeys preferred the static task over the other two tasks (Fig. 1), and that the probability of selecting the static task increased and the dynamic task decreased across sessions (Table 1; Fig. 2). Interestingly, the position of the screen buttons (which changed every hour) did not substantially influence the monkey's

probability to select the static task but had an effect for the picture and dynamic tasks (Table 1; Fig. 3). Monkeys were thus more likely to select the picture task when the stimulus was positioned on the right and more likely to select the dynamic task when the stimulus was positioned in the middle (Fig. 3). We found little evidence that time of day influenced the monkeys' preference of the three MCI tasks (Table 1).

Table S1: Output of the Bayesian generalized linear mixed model investigating task preference during the Multiple Choice Interface experiment.

	Estimate	SD	Lower CI	Upper CI	Pr
**Static**					
Intercept	1.48	0.44	0.56	2.28	1.00
Screen button position (middle)[a]	0.63	0.41	-0.19	1.41	0.94
Screen button position (right)[a]	-0.61	0.37	-1.29	0.16	0.95
Time of day[b]	0.10	0.40	-0.68	0.88	0.60
Session[b]	0.40	0.22	-0.07	0.81	0.96
**Dynamic**					
Intercept	0.39	0.29	-0.19	0.97	0.91
Screen button position (middle)[a]	0.81	0.34	0.13	1.45	0.99
Screen button position (right)[a]	-0.46	0.29	-1.01	0.14	0.93
Time of day[b]	-0.23	0.42	-1.07	0.58	0.72
Session[b]	-0.28	0.15	-0.59	0.00	0.98

Estimate: slope of the predictor; SD: standard deviation of the estimate; CI: 95 % credible interval; Pr: proportion of the posterior samples that fall on the same side of 0 as the mean. [a]Left was the reference level for choice position in both models. [b]Time of day and session were z-transformed.

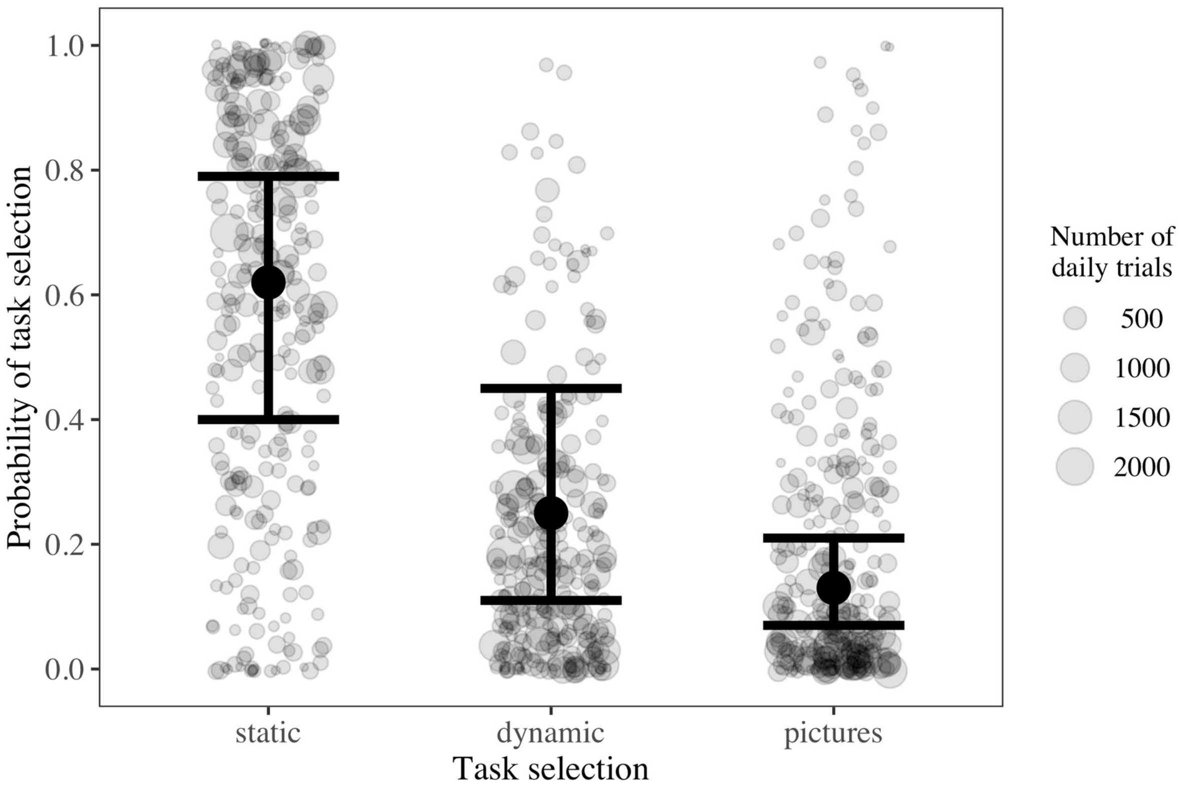

Figure S1: Probability of task selection in the Multiple-Choice Interface experiment. Each dot represents the proportion of trials in which each task was selected by each monkey for each session and screen button position. Solid dots and whiskers indicate the model probability estimates and the 95 % credible intervals, respectively. Model probability estimates and credible intervals calculated from a model run with all covariates at their mean and the screen button position is set to 'middle'. Dot size reflects the total number of trials conducted by each monkey for each selection, session, and screen button position (range: 2 to 2196 trials).

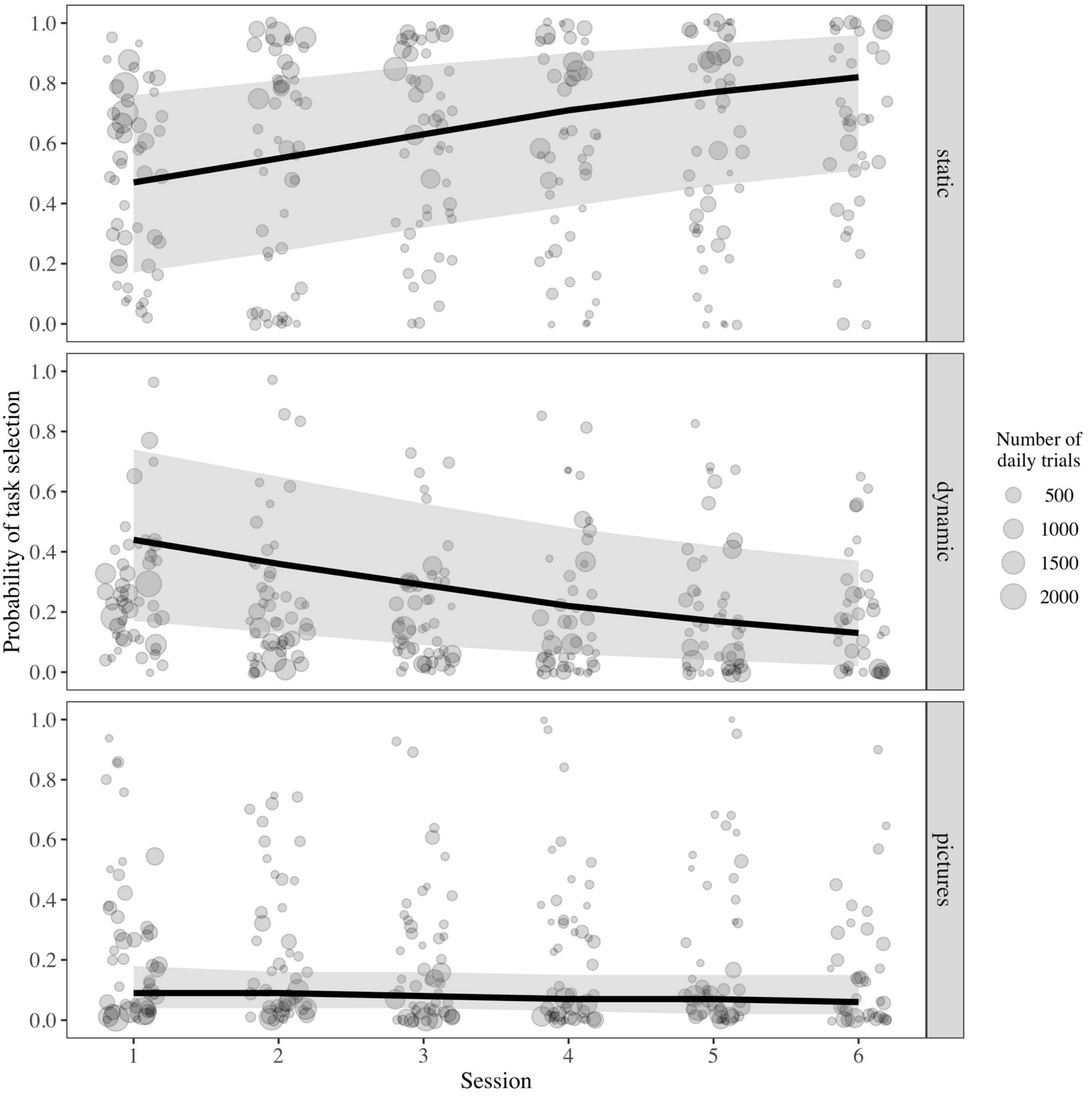

Figure S2: The effect of session on task selection behavior in the Multiple-Choice Interface experiment. Each dot represents the proportion of trials in which each task was selected by each monkey for each session and screen button position. Solid dots and whiskers indicate the model probability estimates and the 95 % credible intervals, respectively. Model probability estimates and credible intervals calculated from a model run with all covariates at their mean and the screen button position is set to 'middle'. Dot size reflects the total number of trials conducted by each monkey for each selection, session, and screen button position (range: 2 to 2196 trials).

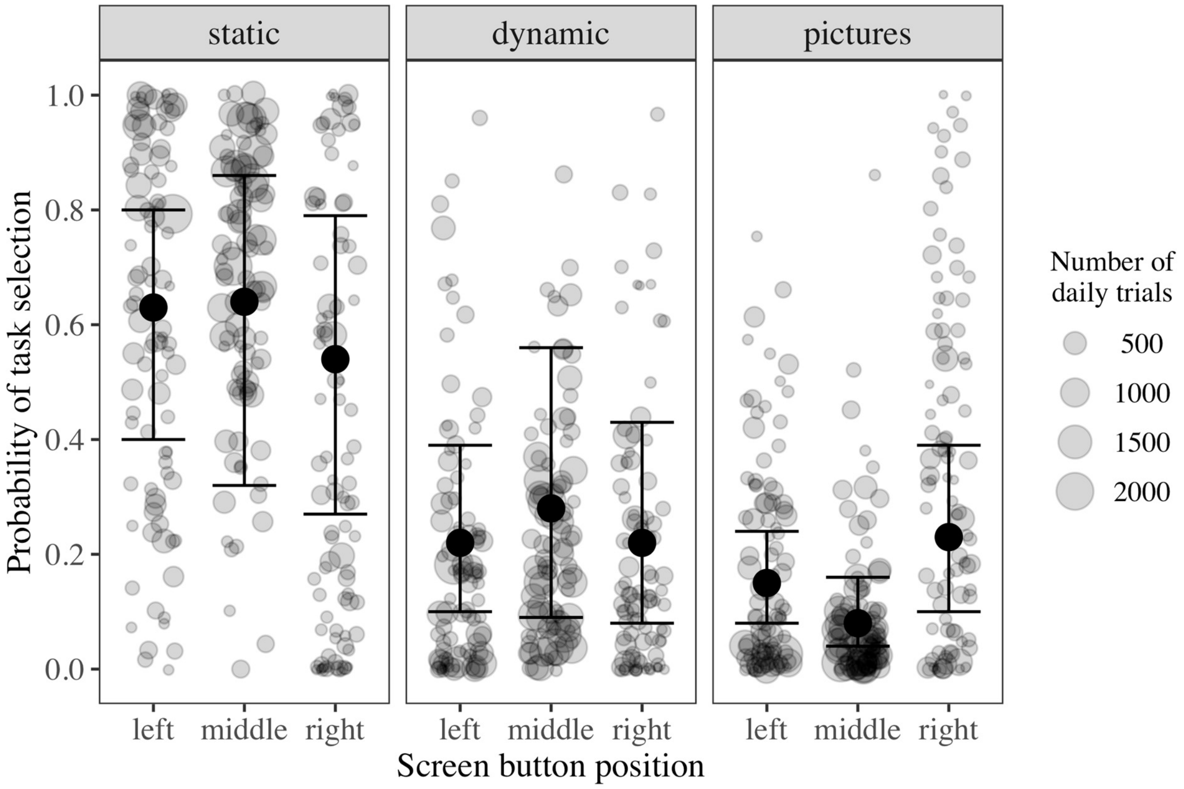

Figure S3: The effect of the screen button position on task selection behavior in the Multiple-Choice Interface experiment. Each dot represents the proportion of trials in which each task was selected by each monkey for each session and screen button position. Solid dots and whiskers indicate the model probability estimates and the 95 % credible intervals, respectively. Model probability estimates and credible intervals calculated from a model run with all covariates at their mean and the screen button position is set to 'middle'. Dot size reflects the total number of trials conducted by each monkey for each selection, session, and screen button position (range: 2 to 2196 trials).
